# Association of type 2 diabetes mellitus and ratio of transmitral E wave velocity to early diastole mitral velocity with cardiovascular events in chronic kidney disease

**DOI:** 10.18632/oncotarget.21768

**Published:** 2017-10-10

**Authors:** Po-Chih Chen, Jiun-Chi Huang, Szu-Chia Chen, Pei-Yu Wu, Jia-Jung Lee, Yi-Wen Chiu, Jer-Ming Chang, Hung-Chun Chen, Yeou-Lih Huang

**Affiliations:** ^1^ Department of Medical Laboratory Science and Biotechnology, College of Health Sciences, Kaohsiung Medical University, Kaohsiung, Taiwan; ^2^ Department of Laboratory Medicine, Kaohsiung Medical University Hospital, Kaohsiung Medical University, Kaohsiung, Taiwan; ^3^ Graduate Institute of Clinical Medicine, College of Medicine, Kaohsiung Medical University, Kaohsiung, Taiwan; ^4^ Division of Nephrology, Department of Internal Medicine, Kaohsiung Medical University Hospital, Kaohsiung Medical University, Kaohsiung, Taiwan; ^5^ Department of Internal Medicine, Kaohsiung Municipal Hsiao-Kang Hospital, Kaohsiung Medical University, Kaohsiung, Taiwan; ^6^ Faculty of Medicine, College of Medicine, Kaohsiung Medical University, Kaohsiung, Taiwan; ^7^ Faculty of Renal Care, College of Medicine, Kaohsiung Medical University, Kaohsiung, Taiwan; ^8^ Department of Internal Medicine, Kaohsiung Municipal Cijin Hospital, Kaohsiung Medical University, Kaohsiung, Taiwan; ^9^ Department of Chemistry, National Sun Yat-sen University, Kaohsiung, Taiwan

**Keywords:** diabetes mellitus, transmitral E wave velocity, early diastole mitral velocity, chronic kidney disease, cardiovascular event

## Abstract

The association between DM and left ventricular diastolic dysfunction, assessed using the ratio of peak early transmitral filling wave velocity (E) to early diastolic velocity of mitral annulus (Ea), with cardiovascular (CV) outcomes in patients with chronic kidney disease (CKD) remains uncertain. This study included 356 CKD stage 3–5 patients underwent echocardiography. All patients were classified into four groups based on the presence of DM and E/Ea ≤ or > 9. CV events included CV death, hospitalization for heart failure, unstable angina or nonfatal myocardial infarction, sustained ventricular arrhythmia, transient ischemic attack, and stroke. There were 58 CV events during the mean observation period of 25.0 months. A combination of the presence of DM and E/Ea > 9 (vs*.* a combination of non-DM and E/Ea ≤ 9) was associated with CV events in unadjusted model (hazard ratio [HR], 6.990; 95% confidence interval [CI], 2.753–17.744; *p* < 0.001), and in a multivariate adjusted model (HR, 3.037; 95% CI, 2.088–7.177; *p* = 0.025). In the patients without DM, the E/Ea ratio (*p* = 0.033) improved the prediction of CV events, compared to the E/Ea ratio (*p* = 0.018), left atrial diameter (*p* = 0.016) and left ventricular mass index (*p* = 0.001) in the patients with DM. The combination of DM and left ventricular diastolic dysfunction was associated with CV events in patients with CKD stage 3–5. Assessments of DM status and E/Ea ratio may facilitate identifying high-risk patient population of unfavorable CV outcomes.

## INTRODUCTION

Chronic kidney disease (CKD) is an emerging healthcare burden, affecting approximately 10% adult population worldwide [1], and is associated with unfavorable outcomes. Notably, cardiovascular disease (CVD) remains the leading cause of disability and mortality in individuals with CKD [2, 3]. This higher risk of cardiovascular (CV) events may be partly attributed to higher rates of traditional risk factors in CKD population, such as aging, hypertension, diabetes mellitus (DM), and dyslipidemia [4]. Whereas functional and structural abnormalities of the heart also play a pivotal role in association of CVD with CKD [5–9], as pressure and volume overload are frequently noted in this patient population [10, 11].

The majority cause of CKD is type 2 DM, accounting for approximately 45% of incident cases of end-stage renal disease (ESRD) in Taiwan [12]. Type 2 DM, in particular, is associated with an increased risk of congestive heart failure [13, 14]. The main types of cardiac abnormalities in structure and function associated with DM are coronary artery disease and diabetic cardiomyopathy, which is characterized by left ventricular (LV) diastolic dysfunction [15]. Early diastolic mitral velocity (Ea) and the ratio of transmitral E wave velocity (E) to Ea have been reported to be significantly correlated with LV diastolic function and filling pressure [16, 17]. Of note, emerging evidence indicates the prognostic role of E/Ea ratio in overall and CV death in patients with ESRD [18, 19].

Despite these findings, whether or not a combination of the presence of DM and the E/Ea ratio can predict adverse CV outcomes in patients with CKD has rarely been investigated. Therefore, this study aimed to examine whether a combination of DM and E/Ea ratio was independently associated with CV events in CKD stage 3−5 patients. Furthermore, the present study also assessed the incremental value of echocardiographic parameters in predicting CV events.

## RESULTS

A total of 356 patients with non-dialyzed CKD stage 3−5 were included in this study. 224 (62.9 %) patients were men. There were 139 (39.0%) patients with CKD stage 3, 105 (29.5%) with CKD stage 4, and 112 (31.5%) with CKD stage 5. The mean age was 66.3 ± 12.2 years. The underlying causes of CKD included diabetic kidney disease (n = 194, 54.5%), non-diabetic glomerular diseases (n = 99, 27.8%), tubulointerstitial diseases (n = 37, 10.4%), hypertension (n = 17, 4.8%), and other diseases (n = 9, 2.5%). These 356 study patients were then stratified into four groups based on the presence of DM and median values of E/Ea as follows: DM(-)E/Ea≦9 (n = 96), DM(+)E/Ea≦9 (n = 82), DM(-)E/Ea > 9 (n = 52) and DM(+)E/Ea > 9 (n = 126).

**Figure 1:**
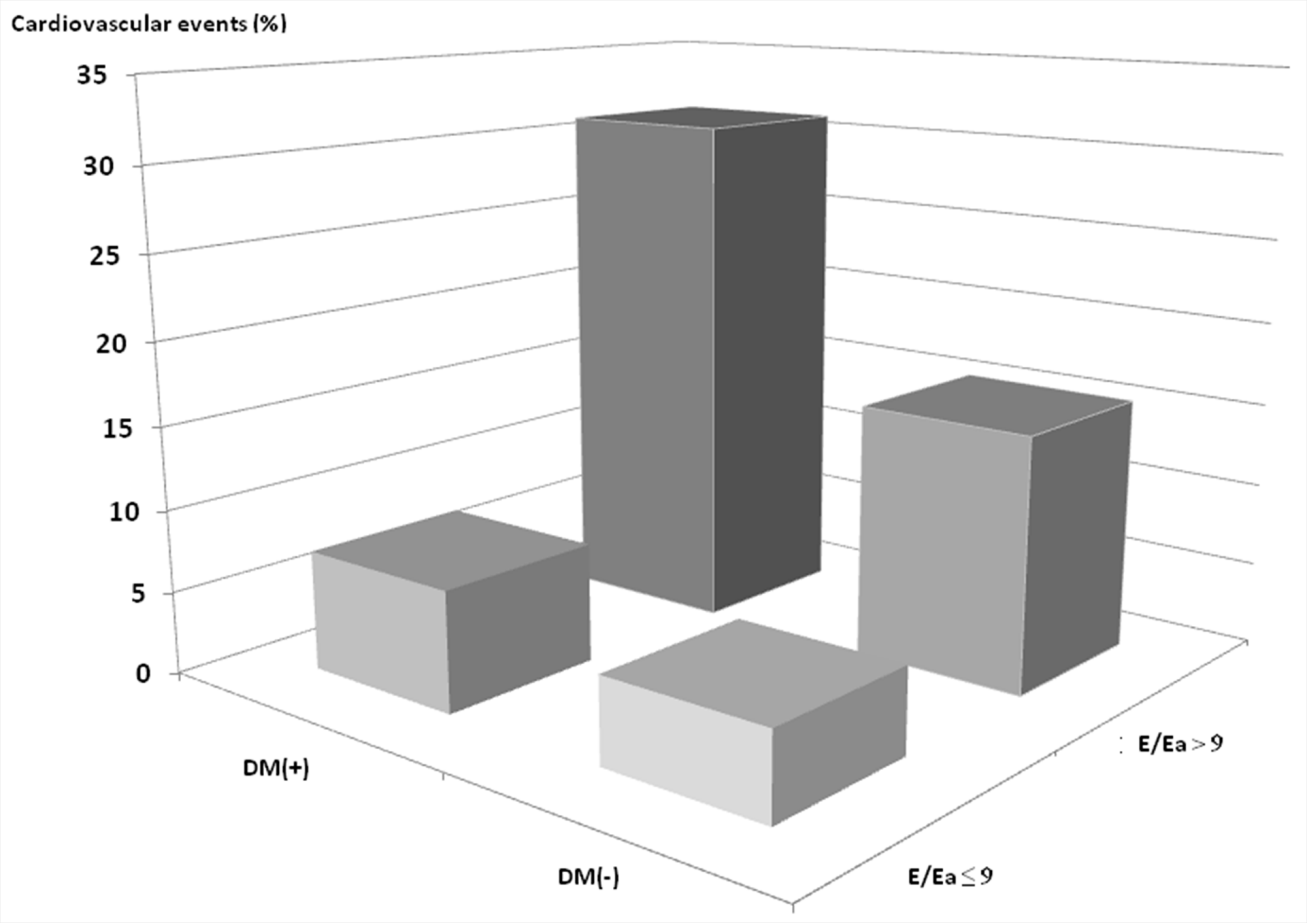
Prevalence of cardiovascular events among 4 study groups There was a significant trend for a stepwise increase in cardiovascular events (5.2%, 7.3%, 15.4% and 31.0%, respectively; *p* < 0.001 for trend) among 4 study groups.

Comparisons of clinical and echocardiographic characteristics among the four study groups are summerized in Table 1. Compared to the DM(-)E/Ea ≤ 9 group, the DM(+)E/Ea > 9 group were older, and had higher prevalence of hypertension, coronary artery disease and proteinuria, higher systolic blood pressure, higher BMI, higher levels of fasting glucose, triglycerides, calcium-phosphorous product and uric acid, and lower levels of hemoglobin and albumin, and a lower estimated glomerular filtration rate (eGFR). Furthermore, the DM(+)E/Ea > 9 group had a higher left atrial (LA) diameter, LV mass index (LVMI), and E/Ea, higher prevalence of LV hypertrophy (LVH) and LV ejection fraction (LVEF) < 50%, and a lower LVEF. Figure 1 demonstrates the prevalence of CV events among the four study groups. There was a significant trend of stepwise increases in CV events among the four groups (5.2%, 7.3%, 15.4% and 31.0%, respectively; *p* < 0.001 for trend).

**Table 1:** Clinical characteristics of patients among study groups

Characteristics	DM(-)E/Ea≦9 (n = 96)	DM(+)E/Ea≦9 (n = 82)	DM(-)E/Ea > 9 (n = 52)	DM(+)E/Ea > 9 (n = 126)
Age (year)	63.8 ± 12.3	63.3 ± 12.6	71.2 ± 12.6^*†^	68.1 ± 10.9^*†^
Male gender (%)	69.8	70.7	59.6	54.0
Smoking habit (%)	28.1	31.7	40.4	31.0
Hypertension (%)	74.0	84.1	86.5	89.7^*^
Coronary artery disease (%)	6.3	6.1	9.6	20.6^*†^
Cerebrovascular disease (%)	8.3	20.7	7.7	19.8
Systolic blood pressure (mmHg)	134.2 ± 19.5	139.7 ± 19.3	145.3 ± 19.4	150.1 ± 23.4^*†^
Diastolic blood pressure (mmHg)	79.4 ± 12.5	80.3 ± 12.3	82.2 ± 12.0	77.1 ± 14.2
Body mass index (kg/m^2^)	24.2 ± 3.6	26.2 ± 4.1^*^	25.6 ± 4.7	26.0 ± 3.6^*^
Laboratory parameters				
Albumin (g/dL)	4.1 ± 0.3	4.1 ± 0.4	4.0 ± 0.4	3.9 ± 0.4^*†#^
Fasting glucose (mg/dL)	98.9 ± 16.9	142.6 ± 62.4^*^	102.7 ± 14.6^†^	151.0 ± 76.6^*#^
Triglycerides (mg/dL)	118 (91.8-170.8)	156 (96.8-238)^*^	132 (83-180)	159.5 (117.5-219.3)^*^
Total cholesterol (mg/dL)	190.5 ± 44.9	195.9 ± 50.7	197.1 ± 39.6	200.9 ± 47.1
Hemoglobin (g/dL)	12.3 ± 2.3	12.4 ± 2.1	11.2 ± 2.2^*†^	10.6 ± 2.0^*†^
eGFR (mL/min/1.73 m^2^)	30.2 ± 15.8	31.1 ± 12.3	20.1 ± 12.0^*†^	21.1 ± 12.5^*†^
Calcium-phosphorous product (mg^2^/dL^2^)	36.3 ± 8.3	37.3 ± 8.4	40.5 ± 9.2^*^	40.3 ± 9.8^*^
Uric acid (mg/dL)	7.8 ± 2.2	7.8 ± 2.4	8.1 ± 1.9	8.7 ± 2.3^*†^
Proteinuria (%)	50.5	61.0	73.1^*^	78.6^*†^
Echocardiographic measurements				
LA diameter (cm)	3.5 ± 0.6	3.7 ± 0.6	3.8 ± 0.6^*^	4.0 ± 0.6^*†#^
LVMI (g/m^2^)	126.4 ± 44.5	119.3 ± 38.2	140.7 ± 46.0	155.3 ± 50.3^*†^
LVH (%)	42.7	39.0	61.5	72.2^*†^
LVEF (%)	70.0 ± 8.3	68.8 ± 9.7	69.8 ± 8.5	65.4 ± 13.3^*^
LVEF < 50% (%)	1.0	2.4	3.8	10.2^*^
EDT (ms)	228.2 ± 68.7	223.0 ± 68.0	228.0 ± 65.7	220.6 ± 63.6
E/Ea	6.7 ± 1.5	7.1 ± 1.4	13.2 ± 4.6^*†^	14.0 ± 4.6^*†^
Cardiovascular events (%)	5.2	7.3	15.4	31.0^*†#^
Duration of follow-up (months)	28.5 (16.4-35.6)	26.8 (17.4-34.4)	29.9 (16.3-37.2)	21.5 (12.9-35.2)

Abbreviations. eGFR, estimated glomerular filtration rate; LA, left atrial; LVMI, left ventricular mass index; LVH, left ventricular hypertrophy; LVEF, left ventricular ejection fraction; EDT, E-wave deceleration time; E, peak early transmitral filling wave velocity; Ea, early diastolic velocity of lateral mitral annulus.

^*^p <0.05 compared with DM(-)E/Ea≦9; ^†^*p* < 0.05 compared with DM(+)E/Ea≦9; ^#^*p* < 0.05 compared with DM(-)E/Ea > 9.

### Risk of CV events

**Figure 2:**
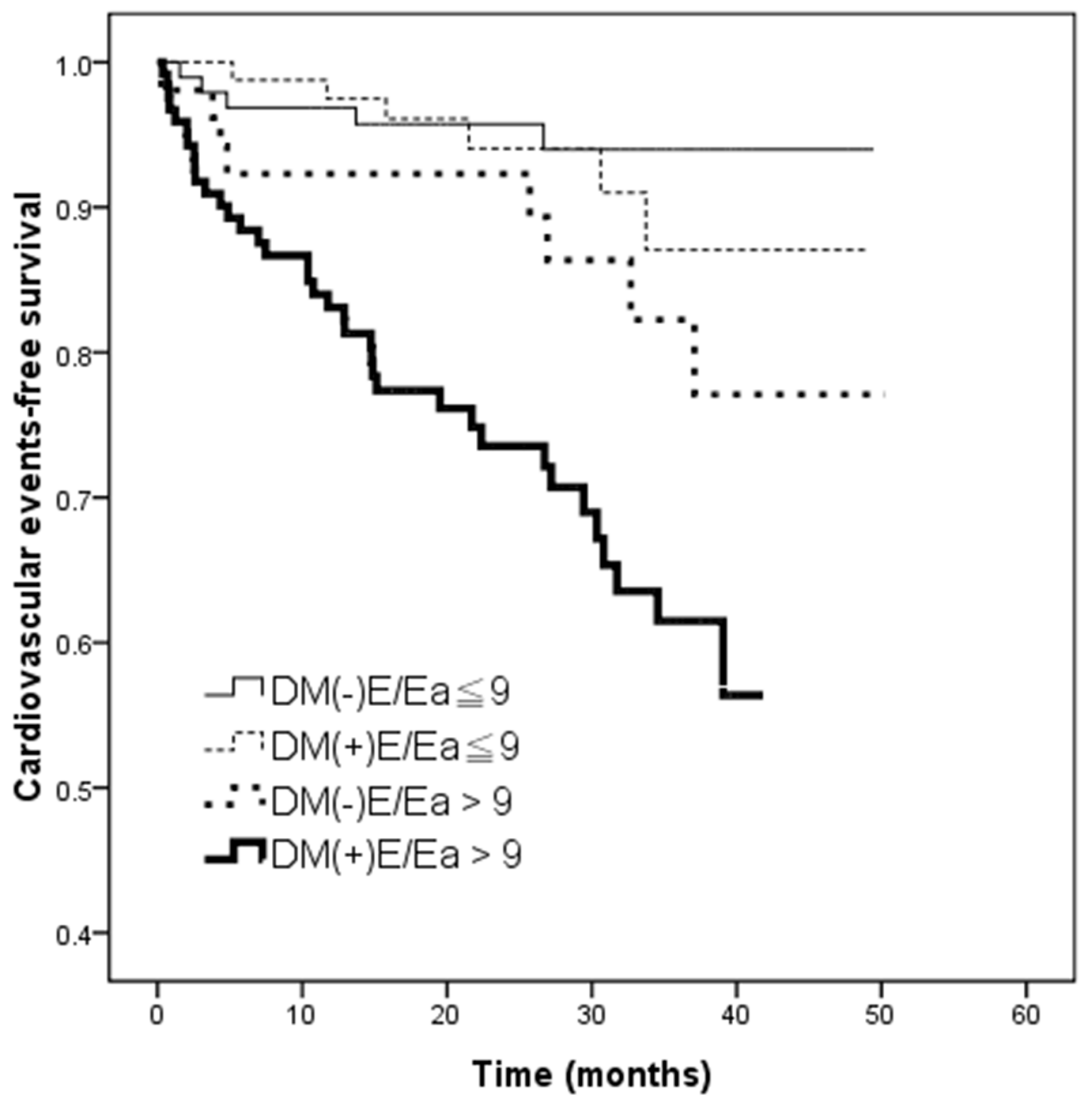
Kaplan–Meier curves for cardiovascular events-free survival (log-rank *p* < 0.001) in patients among 4 study groups The group with DM(+)E/Ea > 9 had a worse cardiovascular events-free survival compared with the group with DM(-)E/Ea≦9.

The mean follow-up time was 25.0 ± 12.2 months. During this period, 58 (16.3 %) CV events were documented, including 16 CV deaths, 11 hospitalizations for unstable angina and nonfatal myocardial infarction, 6 hospitalizations for sustained ventricular arrhythmia, 14 hospitalizations for congestive heart failure, and 11 for transient ischemia attack or stroke. Figure 2 illustrates the Kaplan-Meier curves for CV event-free survival among the four study groups (log-rank *p* < 0.001). Notably, the DM(+)E/Ea > 9 group had the lowest CV event-free survival probability.

Table 2 lists the unadjusted and multivariate adjusted hazard ratios (HRs) of the four groups for CV events. The DM(+)E/Ea > 9 group (*vs.* the DM(-)E/Ea ≤ 9 group) was associated with CV events in the unadjusted model (HR, 6.990; 95% confidence interval [CI], 2.753−17.744; *p* < 0.001) and in the multivariate model adjusted for age, sex, hypertension, coronary artery disease, and cerebrovascular disease (HR, 5.903; 95% CI, 2.301−15.139; *p* < 0.001). Although attenuated association after further adjustment for systolic and diastolic blood pressures, BMI, albumin, fasting glucose, hemoglobin, log-transformed triglycerides, total cholesterol, calcium-phosphorous product, uric acid, eGFR, and proteinuria, the association of DM(+)E/Ea > 9 with CV events remained significant (HR, 3.037; 95% CI, 2.088−7.177; *p* = 0.025).

**Table 2:** Relation of study groups to cardiovascular events using Cox proportional hazards model

Study groups	Unadjusted		Multivariate adjusted (1)		Multivariate adjusted (2)	
	HR (95% CI)	*p*	HR (95% CI)	*p*	HR (95% CI)	*p*
DM(-)E/Ea≦9	Reference		Reference		Reference	
DM(+)E/Ea≦9	1.458 (0.445-4.780)	0.533	1.437 (0.438-4.710)	0.550	1.588 (0.482-5.228)	0.447
DM(-)E/Ea > 9	2.846 (0.931-8.705)	0.067	2.606 (0.849-7.995)	0.094	1.889 (0.613-5.818)	0.268
DM(+)E/Ea > 9	6.990 (2.753-17.744)	< 0.001	5.903 (2.301-15.139)	< 0.001	3.037 (2.088-7.177)	0.025

Values express as hazard ratios (HR) and 95% confidence interval (CI).

Multivariate model (1): adjusted for age, sex, hypertension, coronary artery disease, and cerebrovascular disease.

Multivariate model (2): model (1) + systolic and diastolic blood pressures, BMI, albumin, fasting glucose, log-transformed triglycerides, total cholesterol, hemoglobin, eGFR, calcium-phosphorous product, uric acid and proteinuria.

Abbreviations as Table 1.

The prevalence of LVEF < 50%, 50-60%, 60-70%, 70-80% and ≧ 80% were 5.1%, 10.1%, 35.7%, 40.5% and 8.7%. Because in advanced systolic heart failure, E/Ea showed a poor correlation with intracardiac filling pressures. Therefore, we further performed the analysis after excluding patients with LVEF < 50% (n = 18). The DM(+)E/Ea > 9 group (*vs.* the DM(-)E/Ea ≤ 9 group) was associated with CV events in the unadjusted model (HR, 6.162; 95% CI, 2.399-15.822; *p* < 0.001) and in the multivariate model adjusted for age, sex, hypertension, coronary artery disease, and cerebrovascular disease (HR, 4.472; 95% CI, 1.693-11.8109; *p* = 0.003). However, after further adjustment for systolic and diastolic blood pressures, BMI, albumin, fasting glucose, hemoglobin, log-transformed triglycerides, total cholesterol, calcium-phosphorous product, uric acid, eGFR, and proteinuria, the association of DM(+)E/Ea > 9 with CV events did not achieve the significance (HR, 2.809; 95% CI, 0.968-8.152; *p* = 0.057).

Table 3 shows the univariate analysis of diabetes and other echocardiographic parameters in predicting CV events. In the unadjusted model, diabetes (*p* = 0.001), high LA diameter (*p* < 0.001), high LVMI (*p* = 0.001), low LVEF (*p* = 0.002) and high E/Ea (*p* < 0.001) were associated with increased CV events, but E-wave deceleration time (EDT) was not (*p* = 0.460).

**Table 3:** Univariate analysis of diabetes and echocardiographic parameters to cardiovascular events using Cox proportional hazards model

Parameters	Unadjusted	
	HR (95% CI)	*p*
Diabetes mellitus	2.789 (1.503-5.176)	0.001
LA diameter (per 1 cm)	2.849 (1.809-4.489)	< 0.001
LVMI (per 1 g/m^2^)	1.012 (1.007-1.016)	< 0.001
LVEF (per 1 %)	0.967 (0.948-0.988)	0.002
EDT (per 1 ms)	0.998 (0.994-1.003)	0.460
E/Ea (per 1)	1.111 (1.071-1.152)	< 0.001

Values express as hazard ratios (HR) and 95% confidence interval (CI).

In CKD patients, renal function is important in prognosis. In the study, eGFR is associated with increased CV events in unadjusted model (HR, 0.962; 95% CI, 0.942-0.982; *p* < 0.001). Besides, eGFR was negatively associated with E/Ea (*r* = -0.317, *p* < 0.001). We further performed multivariate analysis in each CKD stages. However, the DM(+)E/Ea > 9 group (*vs.* the DM(-)E/Ea ≤ 9 group) was not associated with CV events in the multivariate analysis in each CKD stages (all *p* > 0.05).

### Predictive value of echocardiographic parameters for CV events

Table 4 presents the incremental values of the echocardiographic parameters in predicting CV events. An addition of E/Ea (*p* = 0.033) to the model adjusted for demographic, clinical, and biochemical risk factors significantly improved the prediction of CV events in the patients without DM. Moreover, an addition of LA diameter (*p* = 0.016), LVMI (*p* = 0.001), or E/Ea (*p* = 0.018) to the aforementioned model significantly meliorated the prediction of CV events in the patients with DM.

**Table 4:** Incremental values of echocardiographic parameters in prediction of cardiovascular events

Parameters	DM(-)		DM(+)	
	Chi-square change	*p*	Chi-square change	*p*
LA diameter (cm)	0.010	0.920	5.856	0.016
LVMI (g/m^2^)	0.022	0.882	10.091	0.001
LVEF (%)	1.874	0.171	0.017	0.896
EDT (ms)	0.080	0.777	0.625	0.429
E/Ea	4.524	0.033	5.621	0.018

*p* value was based on the incremental value compared with the model adjusted for age, sex, hypertension, coronary artery disease, cerebrovascular disease, systolic and diastolic blood pressures, BMI, albumin, fasting glucose, log-transformed triglycerides, total cholesterol, hemoglobin, eGFR, calcium-phosphorous product, uric acid and proteinuria.

Abbreviations as Table 1.

## DISCUSSION

The present study investigated the impact of a combination of the presence of DM and E/Ea ratio on prediction of CV events in CKD stage 3−5 patients. Our findings showed that the DM(+)E/Ea > 9 group was significantly associated with an increased risk of CV events compared to the DM(-)E/Ea ≤ 9 group. The E/Ea ratio improved the prediction of CV events in the patients without DM. Furthermore, LA diameter, LVMI as well as the E/Ea ratio improved the prediction of CV events in diabetic patients with CKD.

The first important finding of the present study is that a combination of the presence of DM and a high E/Ea ratio increased the risk of CV events in CKD stage 3−5 patients. The role of advanced glycation endproducts (AGEs) has been proposed in the pathophysiology of heart failure in diabetic patients [20]. AGEs can impair both diastolic and systolic LV function directly through cross-linkage with extracellular matrix protein or altered calcium handling, and indirectly through interactions with cardiac AGE receptors [21]. In turn, this can lead to low-grade inflammation, increased oxidative stress and altered gene expressions [20, 22], all of which can have a detrimental effect on LV remodeling. Previous studies have indicated that the E/Ea ratio, an estimate of LV filling pressure by Doppler echocardiography, can be used to predict all-cause death in patients with LV systolic dysfunction and after acute myocardial infarction [23–25]. Our results showed that the combination of DM and E/Ea ratio > 9 was associated with an older age, higher prevalence of hypertension, coronary artery disease, and proteinuria, higher systolic blood pressure and BMI, higher levels of fasting glucose, triglycerides, calcium-phosphorous product, and uric acid, lower levels of albumin and hemoglobin, and lower eGFR, all of which could be associated with poor cardiac and renal outcomes. Even after adjustments for these confounding factors, the combination of DM and E/Ea ratio > 9 was still associated with an increased risk of CV events. Therefore, the combination of DM and E/Ea ratio appears to be a useful indicator of unfavorable CV outcomes in patients with moderate to advanced dysfunction of the kidneys.

The second important finding of this study is that LA diameter and LVMI improved the prediction of CV events in diabetic patients with CKD. An enlarged left atrium has been reported to be an indicator of poor CV prognosis including stroke, heart failure, atrial fibrillation, and CV death in a variety of pathologic conditions [26–28], and both volume and pressure overload can subsequently result in LA enlargement. A study on patients with ESRD also reported that an increased LA volume could predict all-cause and CV death in patients undergoing peritoneal dialysis [29]. Moreover, patients with CKD have a high rate of LVH, which can be detected in the early stages of the disease and is associated with older age, anemia, hypertension and poor renal function [11]. Cardiac abnormalities in structure and function have frequently been reported in patients with CKD due to overload in volume and pressure [10, 11]. LVH has also been independently associated with the risk of subsequent CV events in patients with CKD [11]. Carlos et al. evaluated 243 pre-dialysis CKD stage 1−5 patients older than 60 years, and found associations between a higher systolic blood pressure and LVH and CV events [30]. Our findings demonstrated that LA diameter and LVMI improved the prediction of CV events in the patients with DM. This might imply that LA enlargement and LVH can also predict adverse CV outcomes in diabetic patients with CKD stage 3−5.

A decrease in LV systolic function has been reported to predict poor CV prognosis in the general population and in heart failure patients [8, 31–33]. However, in the present study, LVEF did not provide important information with regards to an increased risk of CV events, regardless of the presence of DM. Preserved LV systolic function is found in approximately 50% of heart failure patients, and this has been attributed to diastolic dysfunction [15]. Certain conditions are known to predispose patients to diastolic dysfunction, in particular diabetes [15]. Diastolic dysfunction has been recognized to be more severe in diabetic patients than in those without diabetes [34], and it is also linked to a worse prognosis in these patients [35]. A prevalence rate of 25% to 75% of LV diastolic dysfunction has been reported in diabetic patients [36–40], compared to 5% to 21.8% for LV systolic dysfunction [36, 40, 41]. Therefore, LV diastolic function appears to be a more useful predictor of CV events than LV systolic function.

The present study, however, has several limitations. First, the limited number of CV events debilitates the power of the results. Second, the study patients had CKD stage 3−5; thus, these findings cannot be generalizable to overall CKD populations. Third, treatment with antihypertensive drugs can influence the LV functional parameters and geometry. However, due to ethical concerns we did not withhold any medications in this study. Fourth, in CKD patients, renal function is important in prognosis, although eGFR is put into the multivariate analysis, but coufounding could not be totally eliminated. We further performed multivariate analysis in each CKD stages, but the group of DM(+)E/Ea > 9 was not associated with CV events in the multivariate analysis in each CKD stage. The results may be due to small sample size in each stage, and may be due to other uremia associated risk factors in advanced CKD. Finally, fluid overload is a common problem in patients with CKD and can result in increased CV morbidity and mortality [42]. However, in our study, we lack the volume status data (such as whole-body spectroscopy data) of the study patients. Therefore, we could not exclude the influence of volume ststus on the present findings.

In conclusion, this study showed that the coexistence of DM and LV diastolic dysfunction was an important and independent risk factor for CV events in CKD stage 3−5 patients. Assessments of DM status and E/Ea ratio by echocardiography may facilitate identifying patients with CKD at high-risk of unfavorable CV outcomes.

## MATERIALS AND METHODS

### Study subjects

We conducted this study at a regional hospital in Taiwan. This is a retrospective cohort study. Study patients with CKD stage 3−5 were recruited from the outpatient department during January 2007 to May 2010. CKD was defined on the basis of the Kidney Disease Improving Global Outcomes guidelines [43]. The equation of the 4-variable Modification of Diet in Renal Disease (MDRD) Study was used for calculating eGFR [44]. Based on patients’ baseline eGFR, CKD stages were stratified as stage 3, stage 4, and stage 5 with eGFR of 30−59, 15−29, and < 15 mL/min/1.73 m^2^, respectively. Patients with significant mitral or aortic valvular diseases, atrial fibrillation, and those with poor imaging quality on echocardiography were excluded. Ultimately, 356 CKD stage 3−5 patients were included. The study patients gave written informed consent, and the study protocol was approved by the Institutional Review Board.

### Assessment of cardiac function and structure

Echocardiographic examinations using a VIVID 7 system (General Electric Medical Systems, Horten, Norway) were performed with patient’s lying in a left decubitus posture by two experienced cardiologists. The cardiologists were blind to patients’ clinical information. Images of two-dimensional (2D) and 2D-guided M-mode were obtained from the standard views. The Doppler ultrasound sample volume was placed at the tips of the mitral leaflets and aligned to the flow using an ultrasonic beam. LV inflow waveforms were obtained from apical 4-chamber views. To obtain tissue Doppler images, the sample volume was then placed at the lateral corner of the mitral annulus. The measurements on echocardiography included LA diameter, E, EDT, and Ea. The LV systolic function was assessed using the LVEF. The Devereux equation [45] was applied for calculating the LV mass. The LVMI was defined as the LV mass divided by the body surface area. LVH was defined according to the 2007 European Society of Hypertension/European Society of Cardiology guidelines [46]. All echocardiographic measurements were recorded and offline analyzed using EchoPAC software by another cardiologist.

### Demographic, medical, and laboratory data collection

Through patients’ interview and their medical records, we collected patients’ medical and demographic information, including gender, age, smoking habit (ever or never), and cormorbidities. Overnight fasting blood samples of study patients were measured for laboratory tests. The compensated Jaffé method was used for measurement of serum creatinine levels. Proteinuria with a test result of 1+ or higher by using dipsticks was defined as positive. Study subjects’ urine and blood samples were collected within one month of study enrollment.

### Definition of CV events

CV events were verified by the cardiologists from medical records and chart review. CV events were defined as hospitalization for heart failure, unstable angina or nonfatal myocardial infarction, sustained ventricular arrhythmia, transient ischemic attack or stroke, and CV death. All study patients were followed until the occurrence of a first CV event or until February 2011, whichever occurred first.

### Statistical analysis

The statistical analyses were carried out using SPSS version 19.0 (SPSS Inc., Chicago, IL, USA) for Windows. Data are presented as percentages, mean ± standard deviation or median (25^th^−75^th^ percentile). All study patients were stratified into four groups based on the presence of DM and the median E/Ea value of 9. Comparisons among different groups were analyzed using one-way analysis of variance (ANOVA), followed by Bonferroni correction as the post-hoc test. The time to the occurrence of a CV event and the associated risk factors were assessed using a Cox proportional hazards model. The survival curves for CV events were estimated using the Kaplan-Meier method. Associations between the study groups and CV events were assessed using a stepwise procedure with two models. The first model contained age, sex, hypertension, coronary artery disease, and cerebrovascular disease. The second model included systolic and diastolic blood pressures, BMI, albumin, fasting glucose, log-transformed triglycerides, total cholesterol, hemoglobin, eGFR, calcium-phosphorous product, uric acid and proteinuria. A significant improvement in the predictive power of a model was based on the Chi-square value, and *p* values were on the basis of the incremental value compared to the aforementioned model. Relationships between two parameters were assessed using bivariate correlations (Pearson’s correlation). A *p* value less than 0.05 was considered statistically significant.

## Abbreviations

(CKD): chronic kidney disease
(CVD): cardiovascular disease
(CV): cardiovascular
(DM): diabetes mellitus
(ESRD): end-stage renal disease
(LV): left ventricular
(Ea): early diastolic mitral velocity
(E): transmitral E wave velocity
(LA): left atrial
(LVMI): LV mass index
(LVH): LV hypertrophy
(LVEF): LV ejection fraction
(AGEs): advanced glycation endproducts
(EDT): E-wave deceleration time
(2D): two-dimensional
